# Clinicopathological features, morbidity and survival in gastric adenocarcinoma, with a focus on stage III: a retrospective institutional cohort analysis

**DOI:** 10.1590/0102-672020260000037e1966

**Published:** 2026-08-31

**Authors:** Mariana Freire PINTO, Paulo KASSAB, Osvaldo Antonio Prado CASTRO, Anna Clara Hebling MITIDIERI

**Affiliations:** 1 Santa Casa de São Paulo, Faculdade de Ciências Médicas, Deparment of Surgery – São Paulo (SP), Brazil.

**Keywords:** Stomach neoplasms, Neoplasm staging, Survival analysis, Neoplasias gástricas, Estadiamento de neoplasias, Análise de sobrevida

## Abstract

**Background:**

Gastric adenocarcinoma remains one of the leading causes of cancer mortality worldwide, according to the International Agency for Research on Cancer. Stage III, as defined by the 8th edition of the American Joint Committee on Cancer Tumor, Node, and Metastasis (TNM) system, comprises a biologically heterogeneous group, resulting in clinicopathological variations that directly impact prognosis and therapeutic decisions.

**Aims:**

To evaluate the clinicopathological features, surgical morbidity and mortality, and overall survival of patients with gastric adenocarcinoma treated at a tertiary center, and to analyze prognostic differences among stage III subgroups.

**Methods:**

This retrospective cohort study included patients treated between 2008 and 2018. Demographic variables, tumor characteristics, TNM staging (8^th^ edition), type of surgical procedure, complications according to the Clavien-Dindo classification, and 30-day mortality were analyzed. Overall survival was estimated using the Kaplan-Meier method and compared with the log-rank test, with a significance level of 5%.

**Results:**

This retrospective cohort study included patients treated between 2008 and 2018. We analyzed demographic variables, tumor characteristics, TNM staging (8^th^ edition), type of surgical procedure, complications according to the Clavien-Dindo classification, and 30-day mortality. Overall survival was estimated using the Kaplan-Meier method and compared with the log-rank test, with a significance level of 5%.

**Conclusions:**

Stage III gastric adenocarcinoma demonstrates marked clinicopathological heterogeneity, which directly impacts morbidity, mortality, and survival. Lymph node involvement is a relevant prognostic determinant.

## ARTICLE HIGHLIGHTS

Stage III disease was the predominant presentation at diagnosis in this cohort.

Significant prognosis heterogeneity was observed among stage IIIA, IIIB and IIIC subgroups.

Tumor depth and nodal burden were strongly associated with postoperative outcomes and survival.

Survival decreased markedly with advancing stage III subclassification, particularly in stage IIIC disease.

## Summary

**CENTRAL MESSAGE:**

Gastric adenocarcinoma is still predominantly diagnosed at advanced stages. Stage III represents a heterogeneous spectrum of disease and should not be considered a single entity. Survival remains poor and is directly associated with staging, with lymph node burden being a key prognostic factor and outcomes markedly declining in more advanced stages.

**PERSPECTIVES:**

Managing stage III gastric cancer remains challenging due to its prognostic heterogeneity. A clear understanding of clinicopathological differences is crucial for therapeutic success. Minimizing discrepancies between clinical and pathological staging is essential to properly select patients who may benefit from curative-intent treatment.

## VISUAL ABSTRACT

**Figure:**
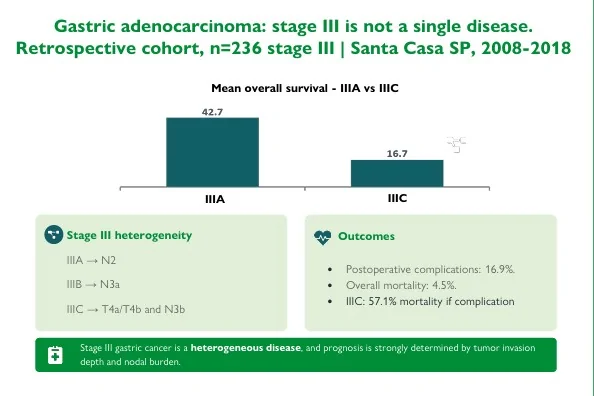

## RESUMO

**Racional:**

O adenocarcinoma gástrico permanece entre as principais causas de mortalidade por câncer no mundo, conforme dados da *International Agency for Research on Cancer*. O estádio III, segundo a 8^a^ edição do sistema TNM da *American Joint Committee on Cancer*, compreende um grupo biologicamente heterogêneo, resultando em variações clínico-patológicas que ditam diretamente o prognóstico e as escolhas terapêuticas.

**Objetivos:**

Avaliar os aspectos clínico-patológicos, a morbimortalidade cirúrgica e a sobrevida global de pacientes com adenocarcinoma gástrico tratados em um centro terciário, além de analisar diferenças prognósticas entre os subgrupos do estádio III.

**Métodos:**

Estudo retrospectivo de coorte incluindo pacientes tratados entre 2008 e 2018. Foram analisadas variáveis demográficas, características tumorais, estadiamento TNM (8ª edição), tipo de procedimento cirúrgico, complicações segundo classificação de Clavien-Dindo e mortalidade em 30 dias. A sobrevida global foi estimada pelo método de Kaplan-Meier e comparada pelo teste Log-Rank, adotando-se nível de significância de 5%.

**Resultados:**

Foram analisados 574 pacientes com adenocarcinoma gástrico, sendo 236 estádio III, correspondendo a 41,1% da amostra total. Observou-se predomínio do sexo masculino (61%) e de tumores localizados no terço distal do estômago (74,6%). Coerentemente, houve progressivo aumento do comprometimento linfonodal do subestádio IIIA ao IIIC, com predominância de N2, N3a e N3b, respectivamente. As complicações pós-operatórias foram significativamente mais comuns no subestádio IIIC. Por fim, a sobrevida média encontrada, conforme esperado, foi inversamente proporcional ao estadiamento, sendo de 42,7 meses no subestádio IIIA e chegando a 16,7 meses no IIIC.

**Conclusões:**

O adenocarcinoma gástrico estádio III apresenta marcada heterogeneidade clínico-patológica, com impacto direto na morbimortalidade e na sobrevida. O comprometimento linfonodal mostrou-se determinante prognóstico relevante.

## INTRODUCTION

 Gastric cancer remains a major global public health problem, ranking fifth in both incidence and mortality worldwide, with a high absolute number of cases due to population aging^4,17^. 

 The global incidence of gastric cancer shows geographic variation, being higher in Asia, South America, and Eastern Europe. In Brazil, most patients with gastric adenocarcinoma are diagnosed at advanced stages, predominantly stage III^21^. Incidence also varies by anatomical location and histological subtype, depending on risk factors. Approximately 30% of cases occur in the fundus and cardia, 30% in the antrum, and 10% present as linitis plastica, with diffuse infiltration of the entire stomach^28^. 

 Gastric adenocarcinoma is a multifactorial disease influenced by environmental and genetic factors. The mean age at diagnosis is in the sixth decade of life, with a male predominance (2:1). Major risk factors include *Helicobacter pylori* infection, dietary habits, obesity, smoking, alcohol consumption, advanced age, socioeconomic status, and genetic predisposition^19^. 

 Although gastric cancer mortality has declined over the past two decades due to sociodemographic improvements and advances in diagnosis and treatment, overall prognosis remains poor, and is directly related to stage at diagnosis^5^. Even after curative-intent treatment, recurrence rates range from 40 to 60%, with 70 to 80% occurring within the first two postoperative years^5^. Delayed diagnosis limits therapeutic options, which are often non-curative^5^. Treatment depends on stage, and early detection is crucial for survival, which is below 10% in metastatic disease^5^. 

 Gastric cancer staging systems have undergone multiple revisions since 2010. The 8^th^ edition of the Union for International Cancer Control/American Joint Committee on Cancer (UICC/AJCC) TNM system^23^ has aligned with the Japanese Gastric Cancer Association classification^14,15^, correcting limitations of the 7^th^ edition^16^. The previous version inadequately distinguished patients with 7–15 positive lymph nodes from those with >15, causing overlap in survival curves between stages IIIB and IIIC. The current edition improved prognostic stratification. In the AJCC 8^th^ edition, T refers to depth of tumor invasion, N to the number of positive regional lymph nodes (N1: 1–2, N2: 3–6, N3a: 7–15, N3b: ≥ 16), and M to distant metastases. Accurate staging is fundamental for defining therapy and estimating prognosis. 

 Stage III is broad, encompassing various combinations of T1-T4b and N0-N3b, constituting a biologically heterogeneous group with significant variations in prognosis and therapeutic response^23^. Even after R0 resection and adequate D2 lymphadenectomy, recurrence rates in stage III remain high, particularly within the first two years, reinforcing the need for multimodal strategies^30^. 

 For T1–2N0 tumors, upfront surgery is usually indicated, whereas T3-T4 and/or N+ tumors are commonly managed with chemotherapy, either neoadjuvant, perioperative or adjuvant^1,15^. 

 The extent of gastric resection depends on location and clinical stage, ranging from endoscopic mucosal resection (EMR) or endoscopic submucosal dissection (ESD) to subtotal or total gastrectomy, distal esophagectomy, or multivisceral resections in selected cases. Although surgery remains the only curative modality, the addition of chemotherapy and/or immunotherapy — neoadjuvant, adjuvant or perioperative — provides a significant survival benefit. A robust randomized phase III trial by Sasako et al. in Japan evaluated 206 patients, and found that stage II and III patients had higher survival rates with adjuvant chemotherapy compared with surgery alone^24^. 

 Prognosis in stage III patients after D2 lymphadenectomy remains heterogeneous. One study of 320 stage III patients undergoing distal or total gastrectomy with D2 lymphadenectomy attributed poor prognosis mainly to lymph node involvement^13^. Another study of clinical stage IIB–III patients concluded that perioperative chemotherapy yields favorable outcomes after radical surgery, with post-neoadjuvant pathological T and N status being the main prognostic factors after R0 resection^11^. In Europe, perioperative chemotherapy is the gold standard for clinical stage II–III, and is recommended by current guidelines^20^. In Japan, upfront surgery followed by adjuvant chemotherapy in selected stage II-III cases is standard^15^. 

 Early gastric cancer, without lymph node or distant metastases, is often asymptomatic and rarely detected outside screening programs. Screening is not widespread, except in high-incidence countries such as Japan, Korea, and Venezuela; thus, diagnosis tends to occur at advanced stages^18^. In Brazil, stage III predominates at diagnosis, and in some centers up to 40% of patients are not surgical candidates^22^. 

 Postoperative follow-up facilitates symptom management, psychological support, and early recurrence detection, although no survival benefit has been proven. Recurrence is most common in the first three years. Little prospective evidence exists regarding the optimal follow-up protocol, which is commonly performed every three months for the first two years and every six months until year 5. Although patients are often considered cured after five years, we advocate at least an annual follow-up indefinitely, as late recurrences have been observed, particularly in patients with a gastric remnant at risk for a second primary malignancy^2^. 

 Clinicopathological studies aimed at deepening knowledge of gastric cancer enable the establishment of a better prognosis and the improvement of therapeutic options^27^. 

 Given the high incidence of stage III gastric cancer in our setting and its heterogeneous prognosis, we analyzed the clinicopathological aspects of these tumors. Therefore, the objective of this study was to evaluate clinicopathological features, surgical morbidity and mortality, and overall survival in patients with gastric adenocarcinoma, with an emphasis on prognostic differences among stage III subgroups according to the 8^th^ edition of the TNM system. 

## METHODS

 We conducted a retrospective cohort study of patients with histologically confirmed gastric adenocarcinoma who underwent surgical treatment with curative or palliative intent at the Gastroesophageal and Bariatric Surgery Unit of Santa Casa de São Paulo from 2008 to 2018. This tertiary referral public center manages patients sent from both public and private health systems. 

 Inclusion criteria: age ≥18 years, histological diagnosis of gastric adenocarcinoma, gastric surgical procedure performed at the institution, available clinical, pathological, and surgical data for tumor classification, and staging updatable to the 8^th^ edition TNM system (Table 1 and Table 2). 

**Table 1:** Pathological staging of gastric cancer.

	N0	N1	N2	N3a	N3b	M1
T1	IA	IB	IIA	IIB	IIIB	IV
T2	IB	IIA	IIB	IIIA	IIIB	IV
T3	IIA	IIB	IIIA	IIIB	IIIC	IV
T4a	IIB	IIIA	IIIA	IIIB	IIIC	IV
T4b	IIIA	IIIB	IIIB	IIIC	IIIC	IV
M1	IV	IV	IV	IV	IV	IV

**Table 2:** Description of the T and N categories of gastric cancer staging.

T	Description	N	Description
Tx	Primary tumor cannot be assessed	NX	Regional lymph nodes cannot be assessed
T0	No evidence of primary tumor	N0	No regional lymph node metastasis
Tis	Carcinoma in situ / high-grade dysplasia (intraepithelial, without invasion of the lamina propria)	N1	1 to 2 regional lymph nodes involved
T1	T1a – invades lamina propria or muscularis mucosae; T1b – invades submucosa	N2	3 to 6 regional lymph nodes involved
T2	Invades muscularis propria	N3	7 or more regional lymph nodes involved
T3	Invades subserosa	N3a	7 to 15 regional lymph nodes involved
T4	T4a – perforates serosa; T4b – invades adjacent structures	N3b	16 or more regional lymph nodes involved

 Exclusion criteria: non-operative management, gastric tumors of other histology (GIST, lymphoma, neuroendocrine tumors), insufficient data for analysis of main variables, and complete loss to follow-up. 

 Data were retrospectively collected from physical and electronic medical records, pathology reports, surgical reports, and outpatient records using a standardized structured form. In cases of discrepancy, pathology reports were prioritized for tumor variables and surgical reports for operative variables. 

 Variables analyzed: sex, age at diagnosis, smoking, alcohol use, tumor location (proximal third, distal third, or whole stomach), Lauren histological subtype, Borrmann macroscopic classification, T category, N category, M status, TNM stage (8th edition) with an emphasis on stage III subgroups (IIIA, IIIB, IIIC), type of gastrectomy (subtotal or total), reconstruction technique, extent of lymphadenectomy, therapeutic intent (curative or palliative), postoperative complications per Clavien-Dindo classification, 30-day/in-hospital mortality, and overall survival. 

 Primary outcome: clinicopathological, surgical, and prognostic characterization of patients with gastric adenocarcinoma, with comparative analysis among stage III subgroups. 

 Secondary outcomes: frequency of postoperative complications, 30-day/in-hospital mortality, distribution of histological subtypes and tumor patterns, and overall survival by stage and stage III subgroup. 

 Postoperative mortality was defined as death within 30 days of surgery or during the same hospitalization. Overall survival was calculated from diagnosis to death from any cause or last follow-up. Patients alive at last visit were censored. 

 To minimize selection and information bias, we included consecutive cases from the study period, used standardized definitions, and reclassified all eligible cases using a uniform TNM system. 

 A convenience sample comprising all eligible patients treated during the study period was used. No prior sample size calculation was performed due to the retrospective and descriptive design. All patients were followed for at least five years postoperatively or until death. 

 Qualitative variables are presented as absolute and relative frequencies. Quantitative variables are expressed as mean±standard deviation or median with interquartile range, according to distribution. Group comparisons used the chisquare test for categorical variables, Student’s t-test for continuous variables between two groups, and variance analysis (ANOVA) for multiple groups. 

 Overall survival was estimated using the Kaplan-Meier method and compared with the log-rank test. Median survival and 1-, 3-, and 5-years estimates were calculated when feasible. The study was conducted in accordance with the Declaration of Helsinki and national regulations for human research. The institutional Research Ethics Committee approved the protocol (Certificate of Presentation for Ethical Appreciation — CAAE: 6.833.537) and waived informed consent due to the retrospective design and use of secondary data. 

## RESULTS

### Overall cohort

 The cohort included 574 patients, predominantly male (61%), and mostly White (60.34%). Among 221 patients with available data, 52% reported regular alcohol consumption; among 222 patients with data on smoking, 58.1% were current or former smokers. 

 Most tumors were located in the distal portion of the stomach (74.6%), followed by the proximal stomach (15.3%), gastric remnant (5.4%), and diffuse involvement of the entire stomach (4.7%). 

 By Borrmann classification, type III tumors predominated (58.16%), followed by type II (22.44%) and linite plastica (11.32%). The Lauren diffuse subtype was most common (59.1%), followed by the intestinal subtype (39.5%). 

 According to the TNM 8^th^ edition, most patients had locally advanced disease, with T4a tumors predominating (37.1%), and no distant metastases in 91.5% (M0). The mean number of resected lymph nodes was 27.57 per patient. Lymph node involvement was heterogeneous: 42.6% had N0 disease, whereas 43.3% had N2 to N3b disease, indicating ≥7 positive nodes. 

 Complete staging showed a predominance of stage III disease (subgroups IIIA, IIIB, and IIIC: 41.1%), followed by stage I (IA and IB: 31%). Stage IV metastatic disease occurred in 8.5%. 

 Regarding surgical treatment, 98.6% of patients underwent gastrectomy, predominantly with curative intent (R0 resection: 71.3%). Subtotal gastrectomy was performed in 74.2%, and total gastrectomy in 24.4%. Endoscopic resection was rare (0.2%). Roux-en-Y was the most common reconstruction (66.1%), followed by Billroth II (28.1%). Table 3 shows the clinicopathological and surgical characteristics and postoperative outcomes of the patients analyzed. 

**Table 3:** Clinicopathological and surgical characteristics and postoperative outcomes (overall sample).

Characteristic		n (%)	Characteristic	n (%)
**Race**		290 (100)	**Tumor depth (T)**	574 (100)
	African descent	84 (28.96)	*In situ* or complete response	5 (0.9)
	Asian	23 (7.93)	1	138 (24.0)
	White	175 (60.39)	2	75 (13.1)
	Indigenous	2 (0.68)	3	113 (19.7)
**Sex**		574 (100)	4a	213 (37.1)
	Female	224 (39.0)	4b	30 (5.2)
	Male	350 (61.0)	**Lymph nodes (N)**	568 (100)
**Tumor location**		574 (100)	0	242 (42.60)
	Distal	428 (74.6)	1	80 (14.08)
	Proximal	88 (15.3)	2	96 (16.90)
	Linitis plastica	27 (4.7)	3a	102 (17.95)
	Gastric stump	31 (5.4)	3b	48 (8.45)
**Lauren**		574 (100)	**Metastasis (M)**	574 (100)
	Diffuse	339 (59.1)	0	525 (91.5)
	Intestinal	227 (39.5)	1	49 (8.5)
	Mixed	8 (1.4)	**Stage**	574 (100)
**Borrmann**		459 (100)	0	5 (0.9)
	I	10 (2.17)	IA	125 (21.8)
	II	103 (22.44)	IB	53 (9.2)
	III	267 (58.16)	IIA	39 (6.8)
	IV	52 (11.32)	IIB	67 (11.7)
	V	27 (5.88)	IIIA	106 (18.5)
**Resection**		574 (100)	IIIB	93 (16.2)
	Endoscopic	1 (0.2)	IIIC	37 (6.4)
	GDP	1 (0.2)	IV	49 (8.5)
	Proximal gastrectomy	6 (1.0)	**Clavien-Dindo**	574 (100)
	Subtotal gastrectomy	426 (74.2)	No complications	501 (87.3)
	Total gastrectomy	140 (24.4)	I	1 (0.2)
**Reconstruction**		573 (100)	II	29 (5.1)
	Ivor-Lewis	3 (0.5)	IIIA	1 (0.2)
	Pean	26 (4.53)	IIIB	8 (1.4)
	Rosanov	2 (0.34)	IVA	6 (1.0)
	Gastric tube	2 (0.34)	IVB	2 (0.3)
	Billroth II	161 (28.09)	V	26 (4.5)
	Roux-en-Y	379 (66.14)		

**Figure 1:**
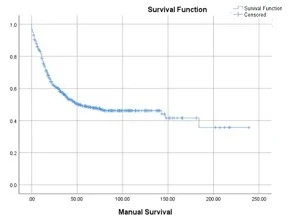
Kaplan-Meier Overall Survival Curve.

 Postoperative complications occurred as follows: Clavien-Dindo grade II in 5.1%, grade III-IV in 3.0%, and grade V (mortality) in 4.5%. During follow-up, 50.2% (n=288) of patients were alive, including seven with disease recurrence. Deaths unrelated to gastric cancer accounted for 7.7% of all deaths. Median overall survival was 53 months (95% confidence interval — 95CI% 11.77–94.22) by Kaplan-Meier analysis (Figure 1). 

### Stage III–specific analysis

 Among the 236 patients with stage III disease, T2–T3 tumors were significantly more frequent in stages IIIA and IIIB (25.42%) than in stage IIIC (1.2%). In contrast, T4a–T4b tumors predominated in stage IIIC (91.9%). T4a was the most common T category across all stage III subgroups. 

 Tumors located in the distal third of the stomach predominated in all stage III subgroups. However, diffuse gastric involvement was more frequent in stage IIIC than in IIIA or IIIB. 

 Borrmann type III tumors were most common in all subgroups. The Lauren diffuse subtype predominated overall, and was most pronounced in stage IIIC. 

 With a mean of >25 resected lymph nodes per case, nodal involvement differed by subgroup: N2 predominated in IIIA, N3a in IIIB, and N3b in IIIC. 

 Substage IIIB had the highest mean intraoperative blood loss (287.1 mL), and the highest packed red blood cell transfusion rate (13.97%). 

 Of 236 stage III patients, 40 (16.9%) had postoperative complications. Seven (17.5%) of these were stage IIIC, and four out of seven (57.1%) died. 

 Mean overall survival was inversely related to substage: 42.7 months in IIIA versus 16.7 months in IIIC (Figure 1). 

## DISCUSSION

 This retrospective study provides a comprehensive clinicopathological characterization of patients with gastric adenocarcinoma treated over a decade at a tertiary center, with an emphasis on stage III disease. The low rate of early tumors and the negligible use of endoscopic resection reflect the absence of effective screening and early detection in our setting. Late diagnosis, high morbidity and mortality, and limited survival remain hallmarks of gastric cancer in Brazil^5,21,22^, consistent with the epidemiological profile of developing countries^21^. 

 The male predominance (61%) aligns with international data reporting a 2:1 male-to-female ration^19^. The higher proportion of White patients (60.34%) reflects the demographics of our catchment area, and does not imply racial predisposition. High rates of smoking (58.1%) and alcohol use (52%) support their role as established risk factors, and highlight the need for primary prevention^19^. 

 Distal tumor location predominated (74.6%) in both the overall cohort and stage III subgroup, consistent with patterns in developing countries. The lower frequency of proximal tumors (15.3%) contrasts with developed Western countries, where obesity and gastroesophageal reflux are more common^19,21,28^. 

 Advanced disease predominated, with a high frequency of T4a tumors (37.1%) and extensive nodal involvement (N2-N3b). These findings partly explain the poor prognosis in stage III. A key finding is the marked prognostic heterogeneity within stage III itself, confirming that this category comprises biologically distinct tumors^15,23^. 

 In stage III, T2–T3 tumors were more common in IIIA and IIIB (25.42%) than in IIIC (1.2%), whereas T4a–T4b tumors predominated in IIIC (91.9%). Notably, a considerable proportion of stage III tumors did not involve the serosa, contrary to expectations. 

 We observed a clear prognostic gradient from IIIA to IIIC, related to both depth of invasion and nodal burden. T1 tumors classified as IIIA or IIIB due to extensive nodal involvement demonstrate aggressive biology with early metastatic spread. Conversely, T4b tumors, even with N0 status, carry a high risk of local recurrence and peritoneal dissemination, justifying their advanced classification. Thus, prognosis in stage III reflects the complex interaction between T and N categories. 

 Nodal burden increased progressively across substages: N2 predominated in IIIA, N3a in IIIB, and N3b in IIIC. This supports lymph node involvement as a major prognostic determinant. Adequate D2 lymphadenectomy is therefore essential for accurate pathological staging and locoregional control. Limited dissections may cause stage migration (Will Rogers phenomenon), leading to inappropriate therapy and worse survival^7^. D2 lymphadenectomy reduces local recurrence and improves survival versus D1 dissection in nodepositive disease, without increasing morbidity or mortality at experienced centers^26^. 

 Morphological features also reflected aggressiveness. Borrmann type III (46.5%) and Lauren diffuse subtype (59.1%) predominated, especially in IIIC. These patterns are associated with deeper invasion, higher risk of peritoneal spread, and poorer treatment response, contributing to unfavorable outcomes. In these cases, multimodal therapy is critical to eradicate micrometastases and reduce recurrence^1,10,15^. 

 Surgically, 98.6% of patients underwent gastrectomy, with R0 resection achieved in 71.3%. Endoscopic resection was rare (0.2%), underscoring the predominance of advanced-stage diagnosis. In stage III, low curative resectability and high morbidity reflect treatment complexity. As an example, stage IIIB had the highest intraoperative blood loss and transfusion rate, suggesting greater technical difficulty or tumor-related systemic effects, corroborating the recent findings of our group^29^. 

 Locally advanced tumors often require extensive or multivisceral resections with a higher complication risk. Surgery alone is rarely sufficient; integration with neoadjuvant, adjuvant, or perioperative chemotherapy is essential to improve oncologic outcomes, as negative margins are critical for survival^1,3,15^. A five-year overall survival in stage III ranges from 20 to 30% in the literature. 

 Regarding reconstruction, Billroth II (BII) was used in a significant proportion because data collection began in 2008. Compared with Roux-en-Y, Billroth II may reduce operative time, with inconclusive evidence on blood loss, but Roux-en-Y is superior for preventing reflux gastritis, esophagitis, dumping syndrome, and delayed gastric emptying^6,12^. There is no evidence of differences in anastomotic leak rates or long-term oncologic outcomes^6,12^. However, gastric stump cancer risk is 2–3 times higher than primary gastric cancer risk, and biliary reflux is a major risk factor, Billroth II carries a risk of remnant gastric cancer after approximately 30 years^8,9^. For this reason, our service does not use BII for curative procedures. 

 Postoperative mortality was highest in stage IIIC. Of 236 stage III patients, 40 (16.9%) had complications; seven (17.5%) were stage IIIC, of whom four (57.1%) died. Although not statistically significant, this high mortality after complications in advanced stages indicates that surgical indication must be carefully weighed. In IIIA, deaths occurred in six of 15 patients with complications (40.0%); in IIIB, in four of 18 (22.2%). These data associate an advanced stage with worse postoperative prognosis. Mean survival was inversely related to substage: 42.7 months in IIIA versus 16.7 months in IIIC. 

 These findings underscore the central role of multimodal therapy in stage III gastric cancer and the need for accurate clinical staging. Perioperative chemotherapy, standard in Western guidelines, is particularly valuable for clinically advanced or borderline resectable tumors, enabling downstaging, higher R0 rates, and early treatment of micrometastases^1,15^. On the other hand, upfront surgery followed by adjuvant chemotherapy may be appropriate in selected cases, with clinical understaging or contraindications to preoperative therapy, such as bleeding, obstruction, or inability to use the enteral route^1,3,10,15^. 

 Limitations of imaging for assessing T category, N status, and occult peritoneal disease make staging laparoscopy with peritoneal cytology essential in stage III^1,15,25^. Although not evaluated here, we recommend staging laparoscopy for all candidates for perioperative or neoadjuvant therapy. 

 Minimizing discrepancies between clinical and pathological staging is critical to select patients who will benefit from curative-intent treatment. Managing stage III gastric cancer remains challenging, and understanding its clinicopathological heterogeneity is crucial for therapeutic success. 

## CONCLUSIONS

 Gastric adenocarcinoma is still predominantly diagnosed at advanced stages in our setting. Stage III represents a heterogeneous spectrum of disease and should not be considered a single entity. Survival remains poor, and is directly associated with substage, with marked decline in more advanced stages. Lymph node burden is a key prognostic determinant. 

## Data Availability

The datasets generated and/or analyzed during the current study are available from the corresponding author upon reasonable request.
